# IL6/CCL2 from M2-polarized microglia promotes breast cancer brain metastasis and the reversal effect of β-elemene

**DOI:** 10.3389/fphar.2025.1547333

**Published:** 2025-05-15

**Authors:** Qian Feng, Cai-Zhi Li, Yi-Hua Zou, Xue-Yu Wang, Xia Yang, Rong Zhang, Zhong-Qiu Liu, Rong-Rong Zhang

**Affiliations:** Guangdong Provincial Key Laboratory of Translational Cancer Research of Chinese Medicines, Joint International Research Laboratory of Translational Cancer Research of Chinese Medicines, International Institute for Translational Chinese Medicine, School of Pharmaceutical Sciences, Guangzhou University of Chinese Medicine, Guangzhou, China

**Keywords:** breast cancer brain metastasis, M2 microglia, IL6 and CCL2 cytokines, IL6-STAT3 signaling pathway, β-elemene

## Abstract

**Introduction:**

Brain metastasis (BM) is the most common and serious complication of breast cancer (BC). There is significant interest in investigating the crosstalk between BC cells and immune cells. β-elemene is the main pharmacodynamic component of *Curcumae Rhizoma*, a traditional Chinese medicine that is commonly used for the clinical treatment and prevention of various tumors. However, the specific underlying mechanism of β-elemene in BC-BM is still unclear.

**Methods:**

An intracardiac (ICT) injection model was used to establish specific BC-BM cells, then an intracarotid (ICD) injection model was used to verify the inhibitory effect of β-elemene in BC-BM. Tumor-cell-conditioned media, a primary microglia co-culture model, and an *in vitro* recruitment experiment were used to explore crosstalk between BC cells and immune cells. TMT-based quantitative proteomic, ELISA, IF, and other molecular biotechnologies were used to investigate the mechanisms.

**Results:**

The BC-BM cells established in our study not only increased BM rates but also exhibit mesenchymal phenotype and activated the JAK–STAT signaling pathway. Microglia, particularly M2 microglia, were enriched in BM lesions and secreted high levels of both IL6 and CCL2. Hypersecretory IL6 reversed the MET process of BC cells by regulating the JAK2/STAT3 signaling pathway to promote colonization in the brain. Increased levels of CCL2 significantly recruited monocytic myeloid-derived suppressor cells (M-MDSCs) to induce an immunosuppressive brain microenvironment. β-elemene could significantly inhibit BC-BM in mice by regulating the IL6/STAT3 signaling pathway and suppressing the M-MDSC recruitment.

**Conclusion:**

Our work first demonstrated that β-elemene regulated the IL6/STAT3 axis and M-MDSC recruitment to reconstruct immunosuppressive brain microenvironment to suppress BC-BM.

## Graphical Abstract

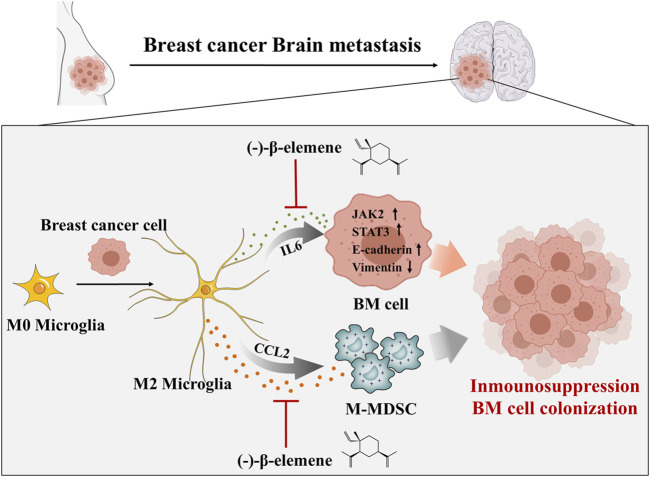

## 1 Introduction

Breast cancer (BC) has become the most prevalent malignancy in women, with 2.3 million new cases and 665,684 deaths reported in 2022 (Bray et al., 2024; Giaquinto et al., 2024). As early diagnosis and therapeutic strategies advance, the overall median survival time of breast cancer patients is greatly extended, but metastasis (especially distant metastasis) remains a considerable threat to the survival of BC patients (Sun et al., 2023). Bone, lung, liver, and brain are common metastatic sites for BC; of these, brain metastasis (BM) is known to occur in 10%–30% of patients and represents the main cause of mortality in patients with BC (Barnholtz-Sloan et al., 2004; Cagney et al., 2017; Lamba et al., 2021). Due to the existence of the blood–brain barrier (BBB), there is no effective clinical treatment for breast cancer brain metastasis (BC-BM) at present; the only available drug is detemozolomide, which has poor efficacy (Bailleux et al., 2021; Brosnan and Anders, 2018). Therefore, it is necessary to illuminate the underlying molecular mechanisms of BC-BM in order to develop novel therapeutic targets, thus helping enhance treatment efficacy and improve the quality of life for BC-BM patients.

The “seed and soil” theory proposed by Stephen Paget in 1889 highlighted that metastasis requires compatible cancer cells (“seeds”) and organ microenvironments (“soil”) (Akhtar et al., 2019; Fidler, 2003). Tumor cells undergo epithelial-to-mesenchymal transition (EMT) to acquire stem-like properties, enabling invasion through basement membranes, intravasation into vasculature, and survival in circulation (Hosonaga et al., 2020; Karimpour et al., 2021). Upon reaching distant organs like the brain, these cells undergo mesenchymal-to-epithelial transition (MET) to extravasate, colonize, and form metastatic lesions. This dynamic interplay between cancer cell plasticity (EMT/MET) and organ-specific microenvironments drives metastatic success (Dongre and Weinberg, 2019; Huang et al., 2022; Yang et al., 2020). Hence, specific interactions between cancer cells and the brain microenvironment are crucial for the metastatic process and tumor colonization.

Microglia, which are resident macrophages in the brain, represent an integral component of the brain microenvironment, participating in innate immunity processes and maintaining central nervous system homeostasis (Nayak et al., 2014). Previous studies have shown that a large number of activated microglia infiltrated into brain metastatic lesions of patients with BC (Caffarel and Braza, 2022; Feng Y. et al., 2024). There are two distinct activated states: the M1 state (which can be identified with iNOS as a specific biomarker) with pro-inflammatory effect and the M2 state (which can be identified by Arg1 as a biomarker) with anti-inflammatory effect in microglia (Chen et al., 2020; Orecchioni et al., 2019). M1 cells inhibit tumors by releasing cytotoxic factors and imparting phagocytotic functionality (Liu et al., 2021). In contrast, M2 cells have a pro-tumor response by activating immunosuppressive factors (Xu et al., 2023). Hence, M2 microglia could inhibit local immunity and then promote metastatic cancer cells to colonize in the brain. However, it is currently unclear whether the process of brain metastatic cells escape immune attack by microglia and are colonized.

*Curcumae Rhizoma* (EZ) is a commonly used traditional Chinese medicine (TCM) that can exert a range of pharmacological effects such as antitumor, antiplatelet aggregation, analgesia, and anti-inflammatory (Gao et al., 2022; Kuzminska et al., 2024; Wu and Tong, 2022; Zhou et al., 2016; Zhu et al., 2023). *Curcuma Rhizome* oil (EZO) is the main pharmacodynamic component of EZ and includes monoterpenoids (such as α-pinene and β-pinene) and sesquiterpenoids (such as β-elemene, zedoary alcohol, and zedoary ketone) (Lin et al., 2024; Rathi et al., 2024). β-elemene has been used to clinically treat various malignant tumors, including ovarian, cervical, colorectal, and lung cancers (Chen et al., 2023; Feng Y. W. et al., 2024; Tan et al., 2021; Xie et al., 2020).

To investigate the molecular mechanisms that promote the metastatic colonization and growth of BC cells in the brain, selective BC-BM cells were isolated and purified from mouse models, and an *in vitro* co-culture system was established to investigate the metastatic mechanism. Furthermore, proteomic analysis revealed that the JAK2–STAT3 pathway was altered in highly metastatic cells. In this study, we aimed to elucidate the underlying mechanism of activated M2 microglia promoting breast metastatic cell colonization and growth in the brain, and to identify the effect and mechanism of β-elemene in preventing BC-BM.

## 2 Materials and methods

### 2.1 Cell culture and compounds

Mouse breast cancer 4T1-luciferase (4T1-luc) cells were purchased from BNCC (Beijing, China) and cultured in DMEM (Gibco, Carlsbad, CA, United States), containing 10% fetal bovine serum (FBS, Gibco, Carlsbad, CA, United States) and 2 μg/mL puromycin at 37°C with 5% CO_2_. Human breast cancer MDA-MB-231-luciferase (MDA-MB-231-luc) cells were purchased from OBiO Technology Corp., Ltd. (Shanghai, China) and incubated in L15 (Gibco, Carlsbad, CA, United States) containing 10% FBS and 2 μg/mL puromycin at 37°C with 100% air. Tocilizumab was purchased from PeproTech Inc. (Suzhou, China), IL6 was purchased from GLPbio (CA, United States), anti-mouse/human/rat CCL2 2H5 was purchased from Selleck Chemicals (Shanghai, China), temozolomide (TMZ) was purchased from Sigma-Aldrich (St. Louis, United States), and β-elemene was purchased from Shanghai Aladdin Biochemical Technology Co., Ltd. (Shanghai, China).

### 2.2 Mouse primary microglia cell isolation

The brain tissues of 2- to 3-day-old newborn mice were separated, and the meninges and blood vessels were completely removed using tweezers. Then, the olfactory bulb, cerebellum, midbrain, and hippocampus were discarded, leaving only the cerebral cortex of the mice brain. The cerebral cortex was cut into 1 mm^3^ pieces and digested by 0.25% EDTA-trypsin for 30 min at 37°C and blown once every 10 min. DMEM containing 10% FBS was used to stop digestion and centrifuged at 1,500 rpm for 10 min. Cells were separated by 37% Percoll and 70% Percoll (1:1) mixture and centrifuged at 300 *g* for 40 min. The intermediate cell layer was carefully removed and washed twice with HBSS, then re-suspended in DMEM containing 10% FBS in an incubator. Immunofluorescence of IBA1 was used to detect the purity of isolation primary microglia, and purity higher than 95% was used for subsequent experiments.

### 2.3 Intracardiac animal model

An intracardiac (ICT) injection model was used to study breast cancer (BC) brain metastasis (BM) (Mizutani and Hirata, 2024). All animal experiments were approved by the Animal Care and Use Committee of the Guangzhou University of Chinese Medicine. Female BALB/c mice and BALB/c nude mice (4–6 weeks old, Guangdong Medical Laboratory Animal Center) were housed under normal SPF surroundings at 21°C ± 1°C and 60% ± 5% humidity. We injected 1 × 10^6^ BC cells (4T1-luc cells or MDA-MB-231-luc cells) into the left cardiac ventricle of 4- to 6-week-old female BALB/c or BALB/c nude mice using 26 G needles. The mice were weighed every other day and monitored for neurological symptoms. BM was detected by *in vivo* bioluminescence imaging. In brief, mice were given 100 μL of 1.5% D-luciferin (Biosharp, Anhui, China) by intraperitoneal injection. Imaging was completed with the Xenogen IVIS system (IVIS Lumina XRMS, Series III) coupled to Living Image Acquisition and Analysis software.

### 2.4 Intracarotid animal model

An intracarotid (ICD) injection model (Lim et al., 2022; Zhang et al., 2017) was used to detect the anti-BC-BM effect of β-elemene *in vivo*. In brief, 5 × 10^3^ 4T1-luc cells were injected into the lumen of the carotid artery of 4- to 6-week-old female BALB/c mice using 31 G needles. Mice were divided into three groups and administrated with solvent, TMZ (25 mg/kg), and β-elemene (25 mg/kg) for 2 weeks. Body weight and neurological symptoms were recorded daily. After 14 days’ administration, fluorescence intensity *in vivo* was measured and sacrificed when the body weight of mice was less than 15 g.

### 2.5 Isolation and purification of brain metastatic cells

Brain metastatic tissues were resected under sterile conditions after sacrifice, and then shredded and placed in a 1:1 mixture of 0.2% collagenase IV (Biosharp, Anhui, China) and RPMI-1640 (Gibco, Carls-bad, CA, United States). They were incubated in a humidified incubator at 37°C for 1 h and blown once every 20 min. The cells were then collected by centrifuge at 1,200 rpm for 5 min and resuspended into 0.25% trypsin. After digestion for 10 min, they were collected and resuspended in RPMI-1640 with 10% FBS and 1% penicillin–streptomycin. Finally, the cells were grown and adhered in 25 cm^2^ culture flasks. BM cells (4T1-BM1, MDA-MB-231-BM1) were selected with puromycin to purification before re-introduction into mice to obtain 4T1-BM2 and MDA-MB-231-BM2 cells.

### 2.6 Proteomic analysis

To investigate differences of protein expression in cells with specialized brain metastatisis, 4T1-luc, 4T1-BM1, and 4T1-BM2 cells were identified and quantified. Cells were washed, lysed, collected, and centrifuged at 8,000 *g* for 30 min, then the supernatant was collected as cellular samples. Trypsinization was performed by trypsin at a 1:30 ratio followed by incubation for 18 h at 37°C. Finally, the peptide samples were obtained by lyophilization. The peptides, which were dissolved in 0.1% formic acid, were separated by C_18_-column and an Easy nano LC system and then analyzed by mass spectrometer. Finally, MaxQuant software (version 1.6.5.0) was used to analyze the results.

### 2.7 *In vitro* conditioned medium co-culture models

In order to simulate the interaction between tumor cells and primary microglia during BM, an *in vitro*-conditioned media (CM) co-culture model was established as follows. We collected 4T1-luc cell and MDA-MB-231-luc cell cultured media and added to the pure mouse primary microglia cells for 48 h. After treatment, cell supernatant was collected and the levels of IL-6 and CCL2 were determined *via* the ELISA kit following the manufacturer’s instructions. Meanwhile, the treated primary microglia cells were collected, and we detected ionized calcium-binding adapter molecule 1 (IBA1, #sc-32725, Santa, United States), arginase-1 (Arg1, #93668, CST, United States), and inducible nitric oxide synthase (iNOS, #ab283655, Abcam, United Kingdom) by immunofluorescence, Western blot, and qRT-PCR.

### 2.8 M-MDSC recruitment experiment and flow cytometry

For migration assays, single myeloid-derived suppressor cells (MDSCs) from the spleens of BM-bearing mice were collected, mechanically disaggregated, and filtered over a 70-μm cell strainer. An *in vitro* immune cell recruitment experiment was conducted using 24-well disposable chemotaxis plates (Corning Incorporated, NY, United States). In brief, 5 × 10^5^ MDSCs isolated from BM-bearing mice spleens were loaded into the top compartments. BC cells co-cultured with primary microglia CM were collected, centrifuged, filtered through a 0.22-μm membrane, and added into the bottom compartment. For the inhibitor experiment, CM was first collected and pretreated with 10 ng/mL anti-mouse CCL2 antibody for 30 min at 37°C. The assembled chamber was incubated for 3 h at 37°C. Cells which migrated into the bottom were collected and examined on BD FACSAria II (BD Biosciences) and analyzed using FlowJo version 10.

For the brain immune cell detection assay, brain tissues were collected, added into RPMI-1640, ground, and filtered over a 70-μm cell strainer. Cells were collected and mixed with 30% Percoll, and then 70% Percoll was added to the upper layer gently. Cells were centrifuged at 300 g for 40 min at 4°C. The middle layer was collected and washed three times with HBSS. The cell pellet was washed by resuspending in fresh PBS buffer (0.5% FBS) and filtered through a 70-μm cell strainer. Anti-mouse antibodies were purchased from BioLegend: CD45 (30-F11, FITC), CD11b (M1/70, APC), Ly6C (hk1.4, PE), and Ly6G (1A8, apc-Cy7).

### 2.9 ELISA

*In vitro* primary microglia cytokines of IL6 (PM6000B) and CCL2 (PMJE00B) were performed using an ELISA kit from R&D Systems China Co., Ltd. according to the manufacturer’s protocol. CM samples were diluted 1:10 for IL6 and 1:50 for CCL2.

### 2.10 Immunostaining and hematoxylin and eosin (H&E) stain

The expression of IBA1 in brain metastatic tissues was assayed by IHC. The metastatic brain tissues were subjected to fixing, paraffin-embedding, and sectioning. The paraffin tumor slides were washed with hydrogen peroxide (3%) to inactivate endogenous peroxide activity and incubated with BSA. Then, the slides were incubated with primary antibody IBA1 overnight at 4°C followed by corresponding secondary antibody. Finally, immunocomplexes were stained with DAB (3,3′-diaminobenzidine) and counterstained with hematoxylin. The slides were dehydrated and photographed under a light microscope (Leica, Wetzlar, Germany). H&E staining of brain tissues was conducted *via* standard procedures. In brief, the brain tissues were fixed with 4% PFA for H&E staining. Images from brain tissues slides were detected with a microscope (Leica, Wetzlar, Germany).

Immunofluorescence (IF) was performed following the standard protocol recommended by Cell Signaling Technology Inc. IBA1, Arg1, iNOS, E-cadherin (#3195, CST, United States), vimentin (#5741, CST, United States), IL6 (#12912, CST, United States), and CCL2 (#MA5-17040, Thermo Fisher, United States) primary antibodies; Alexa Fluor^®^ 594-conjugated AffiniPure Goat Anti-rabbit IgG (#111-585-114, Jackson, United States) and Alexa Fluor^®^ 488-conjugated AffiniPure Goat Anti-Mouse IgG (#115-545-003, Jackson, United States) secondary antibodies; and TGF-β1 were purchased from Jackson ImmunoResearch Laboratories Inc. (Pennsylvania, United States). The cells were fixed on coverslips with 4% paraformaldehyde solution for 20 min, and then permeabilized with 0.1% Triton X-100 (#0694, GBCBIO, China) in PBS. After washing three times, the cells were blocked with 2% bovine serum albumin (BSA) for 30 min, and then stained with the primary antibody at 4°C overnight. The cells were washed thrice in PBST, and then incubated for 1 h with secondary antibodies and counterstained with DAPI. Brain tissue slides were permeabilized, blocked, and washed, followed by incubation with primary and secondary antibody solutions and DAPI. Finally, a Leica TCS SP8 confocal microscope (Leica, Wetzlar, Germany) was used to detect cell fluorescence. ImageJ software (Leica LAS X Hardware Configurator, Wetzlar, Germany) was used to analyze the number of positively stained cells and integrated optical density.

### 2.11 Western blotting and real-time qPCR analysis

Cells or tissues were lysed in RIPA buffer (#89900, Thermo Fisher, United States) containing protease and phosphatase inhibitors (BOSTER, Wuhan, China), and then total proteins were extracted *via* centrifugation. Protein quantifications were determined using the BSA Protein Assay Kit (GBCBIO Technologies Inc., China). Approximately 20 μg of proteins were loaded and separated by 10% SDS-PAGE and then electrotransferred to PVDF membranes (Millipore, United States). These membranes were blocked and washed, followed by incubation with Arg1 (#93668, CST, United States), iNOS (#ab283655, Abcam, United Kingdom), CD206 (#24595,CST), E-cadherin (#3195, CST, United States), vimentin (#5741, CST, United States), STAT3 (#12640, CST, United States), JAK2 (#3230, CST, United States), p-STAT3 (#9145, CST, United States), p-JAK2 (#3776, CST, United States), GAPDH (#sc-32233, Santa, United States), and α-tubulin (#sc-5286, Santa, United States) primary antibodies, and Anti-Rabbit IgG (#7074, CST, United States) and Anti-Mouse IgG (#7076, CST, United States) secondary antibody solutions against target proteins overnight at 4°C. Eventually, the membranes were detected using an ECL gel imaging system (Thermo Scientific™ Chemiluminescent Substrate, United States).

The total RNA of cells was extracted using Trizol reagent (Thermo Fisher, United States). A NanoDrop spectrometer was then used to assess the quality and quantity of total RNA samples. The SYBR^®^ Green *Pro Taq* HS Kit (Thermo Fisher, United States) was used to conduct reverse transcription. qRT-PCR was performed using the SYBR^®^ Green *Pro Taq* HS Kit according to the procedure specification. PCR primer sequences were as follows: 5′- TCC​TTC​CTA​CCC​CAA​TTT​CCA-3′ (sense) and 5′- GTC​TTG​GTC​CTT​AGC​CAC​TCC-3′ (antisense) for IL6. 5′- TCT​CGC​CTC​CAG​CAT​GAA​AG -3′ (sense) and 5′- GGC​ATT​GAT​TGC​ATC​TGG​CT -3′ (antisense) for CCL2. 5′- TGC​GCC​ACA​TGA​AAA​CCA​TC-3′ (sense) and 5′- TTG​GGA​GGA​GAA​GGC​GTT​TG-3′ (antisense) for Arg1. 5′- GCCCAGCCAGCCCAAC-3′ (sense) and 5′-TGG​CCT​TGT​GGT​GAA​GAG​TG-3′ (antisense) for iNOS. 5′-AGA​GTG​TTT​CCT​CGT​CCC​GT-3′ (sense) and 5′-ATGAAGGGGTCGTTGATGGC-3′(antisense) for GAPDH. The data were collected and analyzed using QuantStudio™ Design & Analysis Software, with GAPDH as the internal reference gene.

### 2.12 Wound-healing migration assay

A wound-healing migration assay was used to detect the migration ability of BM cells. We seeded 5 × 105 cells/well in six-well plates to grow to a confluent monolayer overnight and scratched them using a 20 μL pipette tip. The cells were then washed with PBS thrice and incubated in serum-free RPMI-1640 medium for 48 h. The width of the cell-free areas was photographed and recorded using an inverted microscope at 0, 12, 24, and 48 h.

### 2.13 Statistical analysis

GraphPad Prism 8.0 software was employed for statistical analysis. All results were presented as mean ± standard deviation (SD). Statistical differences were analyzed by one-way analysis of variance for multiple groups. p < 0.05 was considered statistically significant.

## 3 Results

### 3.1 IL-6 and CCL2 cytokines and anti-inflammatory M2 microglia phenotype were significantly elevated in the BC-BM microenvironment

**FIGURE 1:**
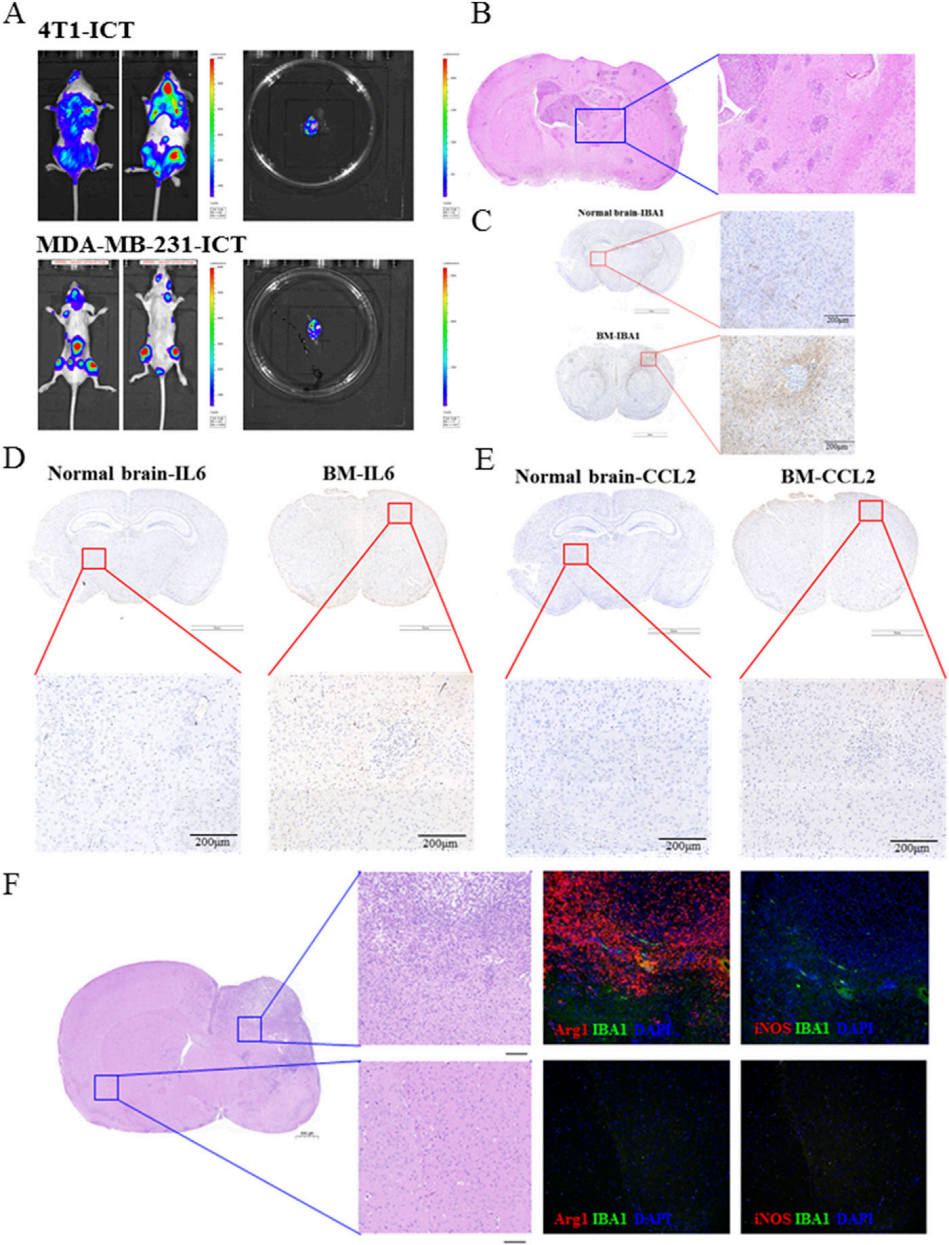
M2 microglia phenotype and IL-6 and CCL2 cytokines were significantly elevated in the BC-BM brain. **(A)** Breast cancer 4T1-luc and MDA-MB-231-luc cells brain metastasis established by intracardiac inoculation. **(B)** HE staining of brain tissue with BC-BM. IHC staining of brain tissue with IBA1 **(C)**, IL6 **(D)**, and CCL2 **(E)**. **(F)** Representative images of immunofluorescence staining, with DAPI (blue) for nuclei, Arg1 (red) for M2-marker, iNOS (red) for M1-marker (magnification: 20×). Upper image was from non-brain-metastatic lesion and lower image was brain-metastatic lesion of mice brain.

To explore the alteration of BC-BM microenvironments, the ICT model was used to establish 4T1-luc and MDA-MB-231-luc brain metastases in mice to simulate the clinical tumor BMs cascade. In brief, 1 × 10^6^ tumor cells were injected into the left ventricle of mice, and the whole-body fluorescence intensity was detected at different times. As shown in Figure 1A, after the injection of 1 × 10^6^ 4T1-luc cells, strong fluorescence could be detected in the whole body of mice at 2 weeks, indicating that 4T1-luc cells had already invaded all systems. After being sacrificed, fluorescence could also be detected in the brain tissue, indicating that BM had occurred in mice. Similar results were obtained in MDA-MB-231-luc cells. HE satin results showed that breast tumor cells had metastasized into the entire brain tissue, with enlarged and deformed nuclei, deep staining, and a clustered arrangement (Figure 1B). In order to investigate the role of the BM microenvironment, especially microglia, the immune cells of brain tissue, in BC-BM brain tissue, we detected the distribution of microglia using IBA-1 IHC. As shown in Figure 1C, compared to normal brain tissue, IBA-1^+^ microglia were enriched in BC-BM brains. Due to the tumor immunosurveillance function of microglia in brain tissue, we then detected the expression of cytokine IL6 and chemokine CCL2 in BC-BM brain tissue. As expected, the expressions levels of IL6 (Figure 1D) and CCL2 (Figure 1E) in BC-BM brain tissue were significantly higher than the expressions levels of those in normal brain tissue. Moreover, we found that in the same brains, Arg1^+^ and iNOS^+^ microglia were significantly clustered at the site of tumor metastasis. Furthermore, Arg1^+^ microglia increased at an overwhelming level compared to iNOS^+^ microglia (Figure 1F). Collectively, these results revealed that in the BC-BM microenvironment, the most dominant cell population is microglia, and particularly Arg1^+^ microglia.

### 3.2 BC-BM cells promoted the secretion of IL-6, CCL2, and M2-type polarization of microglia

**FIGURE 2:**
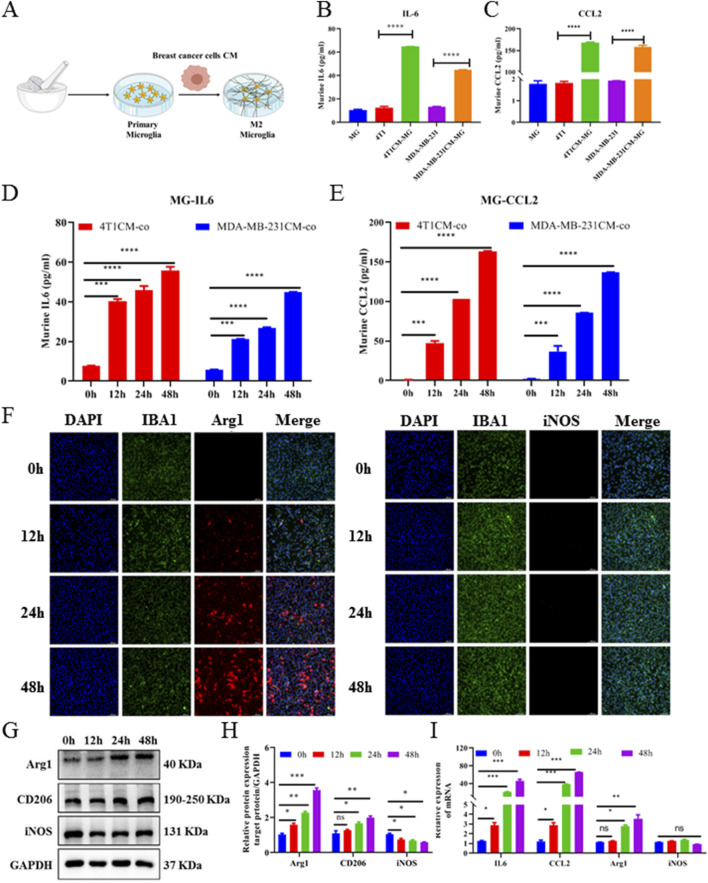
Breast cancer cell CM promoted M2-type polarization, and IL-6 and CCL2 secretion of primary microglia. **(A)** Schematic diagram of *in vitro* CM-co-culture with primary microglia model. ELISA assays of murine-derived IL6 **(B)** and CCL2 **(C)** cytokines from primary microglia cells CM-co-culture model supernatants. Cell supernatants from primary microglia cells treated with CM-coculture medium for 0, 12, 24, and 48 h were collected and measured by IL6 **(D)** and CCL2 **(E)** ELISA assays. **(F)** Representative images of primary microglia cells treated with CM-co-culture medium immunofluorescence staining with DAPI (blue) for nuclei, Arg1 (red) for M2-marker (left image), iNOS (red) for M1-marker (right image) (magnification: 20×). Western blot **(G)** and corresponding gray value analysis **(H)** were used to investigate the expression Arg1, CD206, and iNOS in primary microglia cells treated with CM-coculture medium for 0, 12, 24, and 48 h. **(I)** Relative expression of murine-derived IL6, CCL2, M1-markers (iNOS), and M2-markers (Arg1) mRNA in primary microglia cells treated with CM-coculture medium for 48 h detected by quantitative RT-PCR. GAPDH was used to normalize gene expression. Data are mean ± SD (n = 3). Significant difference versus control group, *p < 0.05, **p < 0.01, and ***p < 0.001.

We next used an *in vitro* CM co-culture assay to further investigate the crosstalk between breast tumor cells and microglia. First, primary microglia (MG) cells were isolated from newborn mice and identified by IBA1 immunofluorescence staining. Then, MG cells were seeded in a 10-cm dish and cultured with BC cell (4T1-luc and MDA-MB-231-luc)-conditioned medium (Figure 2A). ELISA revealed that the levels of cytokine IL6 in MG+4T1-CM and MG + MDA-MB-231-CM were significantly higher than those of cytokine IL6 in the MG or breast cancer cell group (P < 0.001, Figure 2B). Consistently, chemokine CCL2 ELISA results also exerted significantly higher concentrations in the co-culture group than in the single culture group (P < 0.001, Figure 2C). At the same time, concentrations of IL6 and CCL2 in the MG+4T1-CM and MG + MDA-MB-231-CM groups were in a time-dependent manner. The secretion of IL6 and CCL2 from MG were significantly stimulated by BC-CC as early as 12 h co-culture. Compared to MDA-MB-231, CM from mouse 4T1 cells exhibited stronger stimulation for MG, whether in cytokine IL6 or chemokine CCL2 secretion (Figures 2D,E).

To further elucidate the effect of BC cells on the activation of microglia, 4T1-luc conditioned medium (4T1-CM) was collected at different times to stimulate MG. The expression levels of M1 microglia markers (iNOS) and M2 microglia markers (Arg1) were detected using the immunofluorescence assay. As shown in Figure 2F, at 0 h, when MG were not co-cultured with 4T1-CM, the expressions of iNOS and Arg1 were almost undetectable, confirming that microglia were in a quiescent state. After being co-cultured with 4T1-CM, Arg1^+^ microglia were significantly increased in a time-dependent manner, whereas iNOS^+^ microglia had no significant change. The Western blot analysis showed that the protein levels of Arg1 and CD206 in MG were significantly upregulated and the protein level of iNOS had no significant change (Figures 2G, H): Consistently, qRT-PCR results also showed that the expressions of IL6, CCL2, and Arg1 in MG were significantly upregulated after co-culture (P < 0.001, Figure 2I). All results confirmed that BC cells were able to significantly induce M2 polarization of microglia, which was consistent with the *in vivo* brain tissue results. Collectively, these results indicate that BC cells could promote microglial M2-type polarization, as well as increase the secretion of IL6 and CCL2 from microglia.

### 3.3 4T1-BM cells exhibited significant proteomic differences to parental cells

**FIGURE 3:**
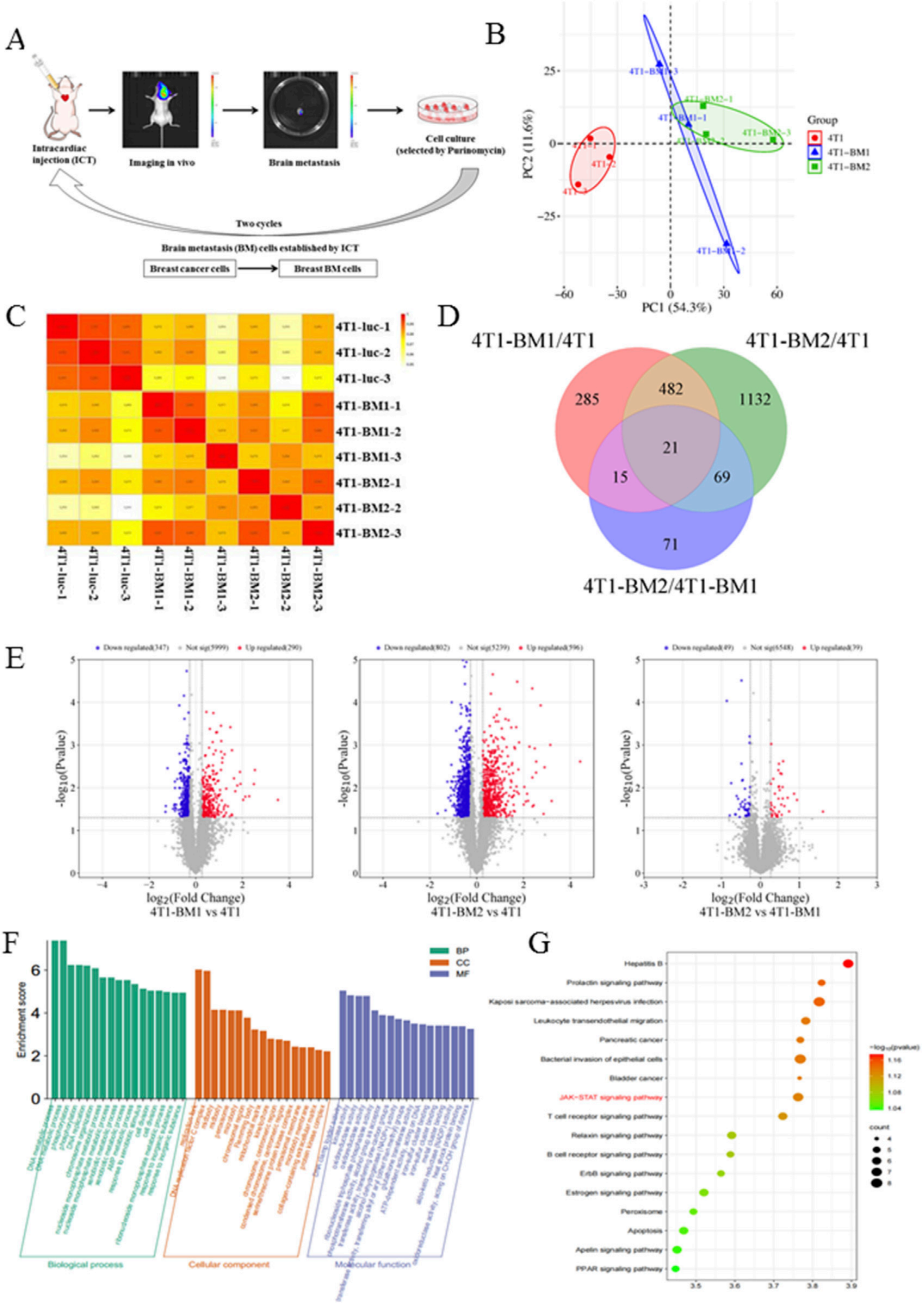
TMT-based quantitative proteomics analysis of 4T1-luc, 4T1-BM1, and 4T1-BM2. **(A)** Schematic representation of *in vivo* selection process. Parent breast cancer cells were inoculated into the left ventricular of BALB/c mice. Tumor cells were isolated from brain lesions and reinoculated after expansion in culture. Cells isolated from the first round of metastases were expanded in culture and reinoculated to obtain second-generation cells (BC-BM2). **(B)** Principal component analysis (PCA) of 4T1-luc, 4T1-BM1, and 4T1-BM2 cells. **(C)** Quantitative protein correlation coefficient distribution analysis of 4T1-luc, 4T1-BM1, and 4T1-BM2 cells. **(D)** Multiple differential expression proteins were displayed as volcano plot. **(E)** Multiple differential expression proteins displayed as volcano plot. **(F)** GO enrichment analysis of differentially expressed proteins. **(G)** KEGG pathway enrichment analysis of differentially expressed proteins.

In order to further analyze the proteomic difference between highly metastatic BC and parent BC, an ICT model was used to enrich BC-BMs. Simply, 4T1-luc cells were injected into mice by ICT; after BMs, tumor cells were separated from mice brain tissue and re-injected into mice. After two rounds of *in vivo* selection, 4T1-BM1and 4T1-BM2 were obtained (Figure 3A). Principal component analysis (PCA) results showed that there were significant differences in protein expression between 4T1-BMs (blue and green) cells and parent 4T1-luc cells (red, Figure 3B). The quantitative distribution of protein correlation coefficients revealed good levels of quantitative repeatability and correlation for all groups (Figure 3C). Compared with the parent cells, in total, 503 proteins in high metastatic cells were changed, of which 285 were significantly upregulated and 218 were significantly downregulated (Figure 3D). As shown in Figure 3E, the horizontal coordinate represents the quantitative ratio of each protein in the comparison group (log2 conversion) and the vertical coordinate represents the P-value of each protein in the comparison group undergoing the T-test (-log10 conversion). Compared with 4T1-luc cells, upregulated and downregulated differential proteins were observed in both 4T1-BM1 and 4T1-BM2 cells. Subsequently, GO enrichment and Kyoto Encyclopedia of Genes and Genomes (KEGG) pathway enrichment analyses of the differential proteins obtained were performed. The functionality of notable differentially expressed proteins predominantly involved biological processes (BP), cell components (CC), and molecular functions (MF) (Figure 3F). Moreover, compared with 4T1-luc cells, the JAK–STAT signaling pathway was significantly upregulated in 4T1-BM cells, which is closely related to signal transduction of various cytokines (Figure 3G).

### 3.4 BC-BM cells revealed a high BM rate and significantly recruited M-MDSCs *in vivo*

**FIGURE 4:**
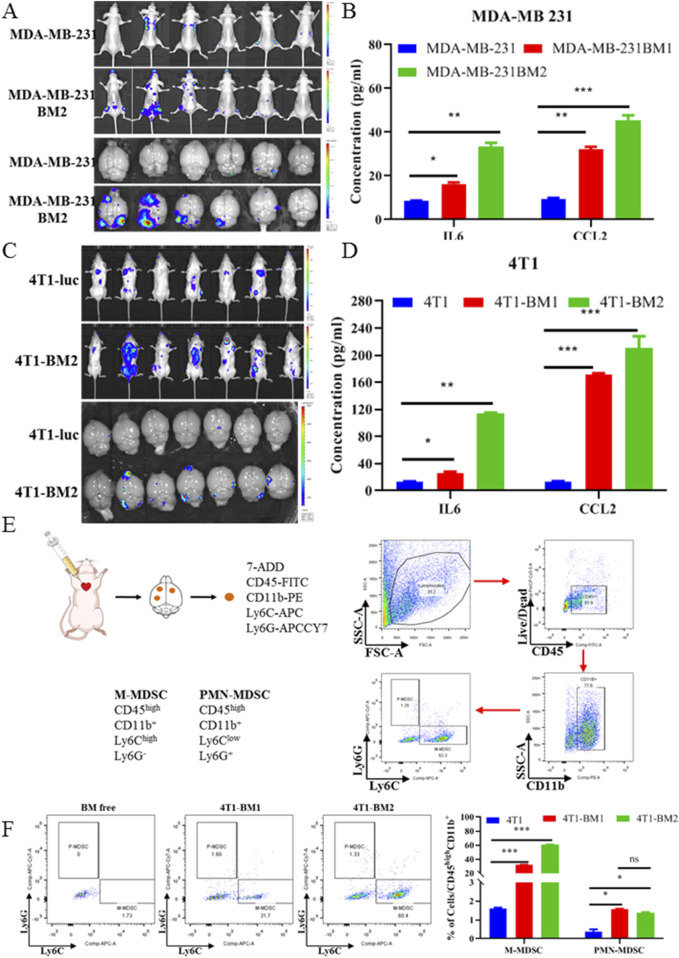
BC-BM cells revealed high BM rate and significantly recruited M-MDSC *in vivo*. *In vivo* and *in situ* fluorescence intensity of human breast cancer parent MDA-MB-231-luc and MDA-MB-231-BM2 cells **(A)**, and mouse breast cancer parent 4T1-luc and 4T1-BM2 cells **(C)**. ELISA assays of murine-derived IL6 and CCL2 cytokines from human breast cancer parent MDA-MB-231-luc and MDA-MB-231-BMs cell supernatants **(B)**, and mouse breast cancer parent 4T1-luc and 4T1-BM2 cell supernatants **(D)**. **(E)** Schematic diagram of immune cells separated from brain tissue and stained with cluster of differentiation markers. **(F)** M-MDSC and P-MDSC distributions in normal mice brain, 4T1-BM1 inoculation mice brain, and 4T1-BM2 inoculation mice brain were detected by flow cytometer. Data are mean ± SD (n = 3). Significant difference versus control group, *p < 0.05, **p < 0.01, and ***p < 0.001.

In order to confirm the *in vivo* BM rate of BC-BM cells after two rounds of *in vivo* selection, the ICT model was first used to compare the *in vivo* BM rate between BC parent cells and BC-BM2 generation cells. As shown in Figure 4A, BM incidence increased from 33.3% for human MDA-MB-231-luc to 66.7% for MDA-MB-231-BM2 *in situ*. Consistently, mouse 4T1-BM2 cells enriched from the ICT model also exhibited a higher *in situ* BM rate (28.57% VS 71.43%, Figure 4C). *In vivo* brain bioluminescence imaging results also confirmed that the incidence of BM in the MDA-MB-231-BM2 group was much higher than that of the MDA-MB-231-luc group (83.3% vs. 33.3%). Mouse 4T1-BM2 cells revealed similar results, with the rate of BM increasing from 42.86% to 100% in the brain (Figure 4C). ELISA results further revealed that the concentrations of IL6 and CCL2 in the supernatants of primary BM cells were significantly upregulated compared with parent cells. The concentration of IL6 in MDA-MB-231-BM2 primary culture supernatant was 33.57 ± 2.12 pg/mL, which was almost four times higher than that of MDA-MB-231 cells (8.61 ± 0.14 pg/mL, Figure 4B). The concentration of CCL2 in MDA-MB-231-BM2 primary culture supernatant was 45.58 ± 3.14 pg/mL, which was almost five times higher than that of MDA-MB-231 cells (9.51 ± 0.63 pg/mL, Figure 4B). Similar results also were observed in mouse 4T1 cells, especially a significantly increased concentration of chemokine CCL2 in 4T1-BM2 primary culture supernatant (13.51 ± 0.78 pg/mL VS 210.58 ± 24.35 pg/mL, Figure 4D). More research has confirmed that chemokine CCL2 can recruit MDSCs and form immunosuppressive microenvironment. Next, we conducted flow cytometry to detect brain MDSCs in 4T1-BM mice and used cell surface markers Ly6C and Ly6G to define different myeloid populations: monocytic MDSCs (M-MDSCs) and polymorphonuclear MDSCs (PMN-MDSCs) (Figure 4E). Our results showed that compared with BM-free brain tissue, both M-MDSCs and P-MDSCs were significantly increased in BM brain tissue, and M-MDSCs were the predominant myeloid population (Figure 4F). In summary, BC-BM cells established by an ICT reinjection model significantly increase not only the rate of brain metastasis but also the M-MDSC population in BC-BM brain tissue.

### 3.5 BC-BM cells exhibited mesenchymal phenotype and activation of JAK2–STAT3 signaling pathway

**FIGURE 5:**
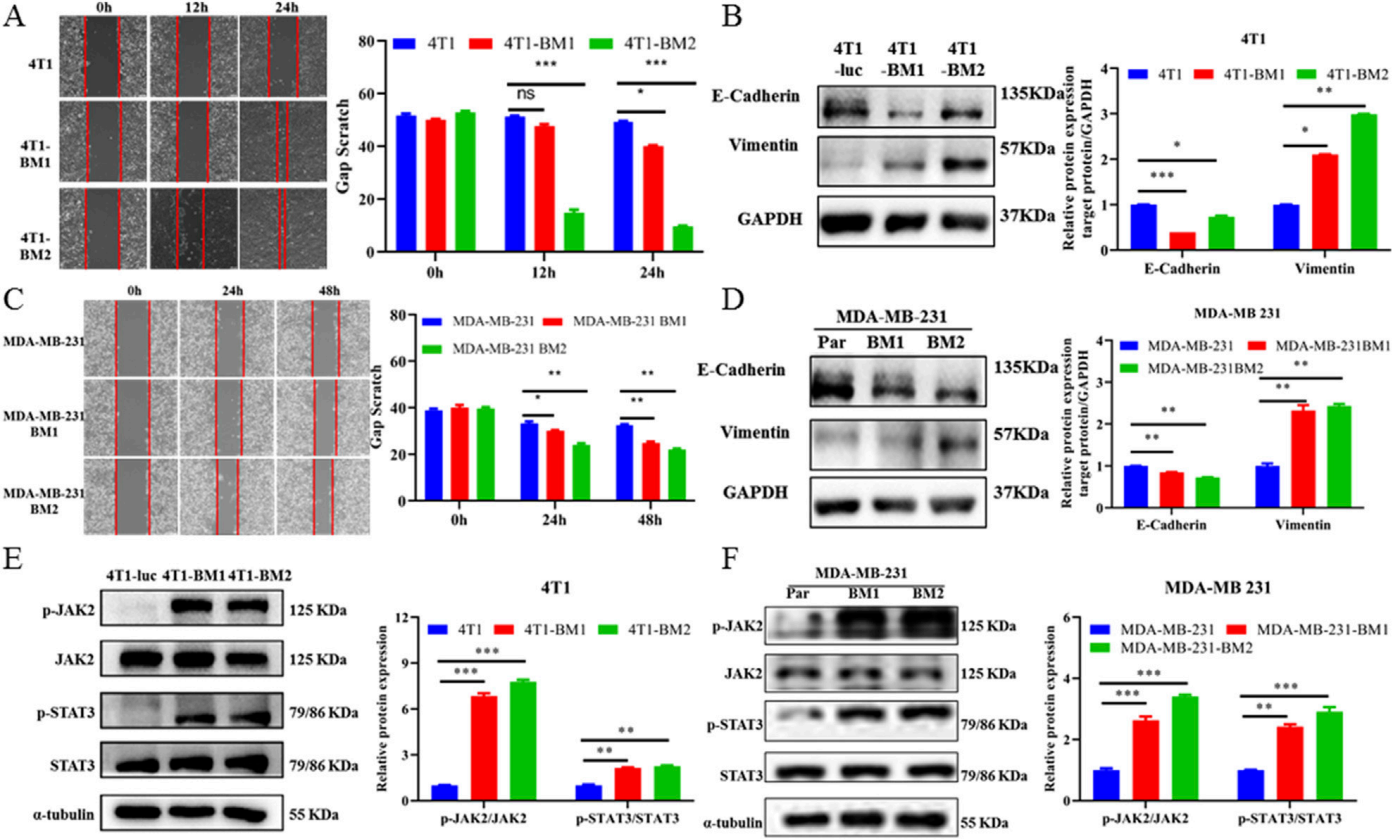
BC-BM exhibited mesenchymal phenotype and activated JAK2-STAT3 signaling. **(A)** Images and statistical analysis of wound healing assay among 4T1-luc, 4T1-BM1, and 4T1-BM2 cells (magnification, 40×). **(B)** Western blot and corresponding gray value analysis of E-cadherin, vimentin in 4T1-luc, 4T1-BM1, and 4T1-BM2 cells. **(C)** Images and statistical analysis of wound healing assay among MDA-MB-231-luc, MDA-MB-231-BM1, and MDA-MB-231-BM2 cells (magnification, 40×). **(D)** Western blot and corresponding gray value analysis of E-cadherin, vimentin in MDA-MB-231-luc, MDA-MB-231-BM, and MDA-MB-231-BM2 cells. **(E)** Protein expressions of p-JAK2, JAK2, p-STAT3, and STAT3 in 4T1-luc, 4T1-BM1, and 4T1-BM2 cells. **(F)** Protein expressions of p-JAK2, JAK2, p-STAT3, and STAT3 in MDA-MB-231-luc, MDA-MB-231-BM, and MDA-MB-231-BM2 cells. Data are shown as mean ± SD (n = 3). *p < 0.05, **p < 0.01, and ***p < 0.001 vs. corresponding parent group.

Increasing evidence has confirmed epithelial mesenchymal transformation (EMT) as a hallmark of cancer metastasis (Dongre and Weinberg, 2019; Huang et al., 2022; Yang et al., 2020). Therefore, a wound healing assay was first used to verify the migration activities among BC parent cells and BC-BM cells. As shown in Figures 5A and C, the cell migratory ability of 4T1-BM2 was significantly enhanced compared with the parent cells after 24 h. MDA-MB-231-BM2 cells also exhibited stronger migratory ability than MDA-MB-231 cells (Figures 5B,D). We next used the Western blot to verify key proteins associated with EMT among BC parent cells and high BM cells. The results showed that as BM increased, the protein expression of epithelial cell marker E-cadherin was significantly decreased in both mouse and human BC cells, whereas the expression of mesenchymal cell marker vimentin was significantly upregulated (Figures 5E,F). These results confirmed that both human and mouse BC-BM cells established by the ICT *in vivo* re-injection model exhibited stronger invasion and metastasis ability than parent cells. Our proteomic results revealed that the JAK–STAT signaling pathway was significantly upregulated in 4T1-BMs cells, and then the Western blot was used to verify key proteins of the JAK–STAT signaling pathway in parent cells and high metastatic cells. Our results showed that phosphorylated JAK2 and STAT3 levels were significantly increased in both 4T1-BMs cells (Figure 5G) and MDA-MB-231-BMs cells (Figure 5H), , which were consistent with our proteomic results. These results indicate that human breast MDA-MB-231-BM2 cells or mouse breast 4T1-BM2 cells exhibited mesenchymal phenotype and activation of the JAK2–STAT3 signaling pathway.

### 3.6 M2-type microglia recruited M-MDSCs to form the brain immunosuppressive microenvironment and promoted brain colonization of BC-BM cells

**FIGURE 6:**
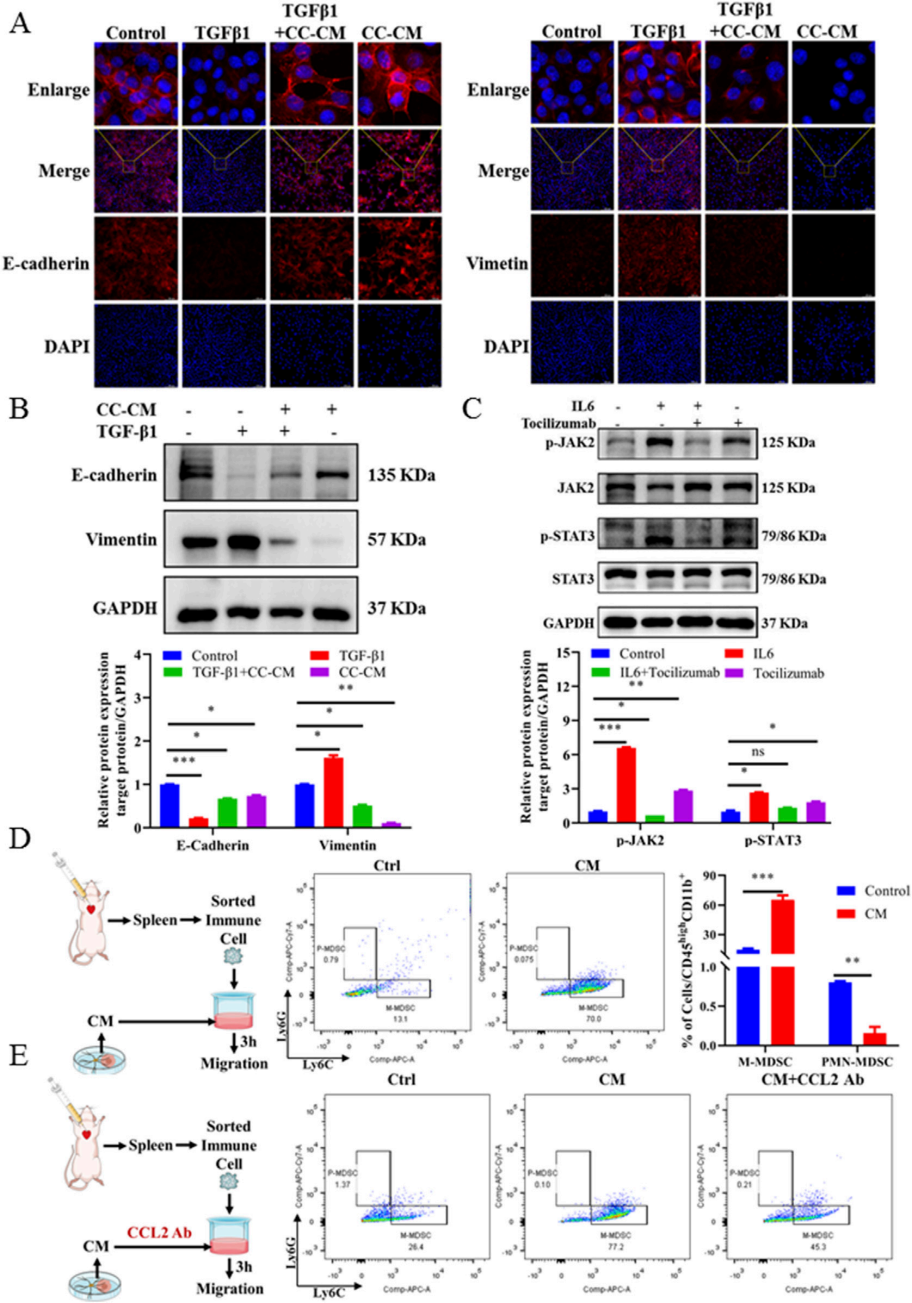
M2-type microglia secrete cytokines that promote the MET process of tumor cells and recruit M-MDSCs. **(A)** E-cadherin (magnification: 400×) and vimentin (magnification: 200×) expression of 4T1-luc cells cultured with different conditions presented by immunofluorescence staining. Blue signal represents DAPI-stained nuclei. Control: normal 4T1-luc cells; TGF-β1: 4T1-luc cells treated with TGF-β1 (2.5 ng/mL) for 24 h; TGF-β1 and CC-CM: 4T1-luc cells treated with TGF-β1 (2.5 ng/mL) and incubated with co-culture CM for 24 h; CC-CM: 4T1-luc cells treated with co-culture CM for 24 h. **(B)** Western blot and corresponding gray value analysis of E-cadherin and vimentin in 4T1-luc cells cultured with different conditions. **(C)** Western blot and corresponding gray value analysis of p-JAK2, JAK2, p-STAT3, and STAT3 in 4T1-luc cells treated with Tocilizumab (2.5 μg/mL), IL6 (200 ng/mL) alone, or IL6-supplemented with tocilizumab. **(D)** Flowchart of *in vitro* MDSC recruitment experiment, flow cytometer detection results, and data statistics diagram. **(E)** Flowchart of *in vitro* MDSC recruitment with anti-mouse/human/rat CCL2 2H5 experiment and flow cytometer detection results. Data are mean ± SD (n = 3). Significant difference versus control group, *p < 0.05, **p < 0.01, and ***p < 0.001.

Based on the above results, we hypothesized that BC cells in brain tissue promoted M2-type polarization of microglia, enhanced secretion of IL6 and CCL2, recruited M-MDSCs to form brain immunosuppressive microenvironment, and promoted brain colonization and the growth of BC-BM cells. We first used the supernatant of activated microglia after co-culture to detect the effect on the EMT process. In brief, we established the EMT process in 4T1-luc cells by using TGF-β1 treatment as previously reported, and then cells were exposed with co-culture conditioned medium (CC-CM) harvested from primary microglia treated with BC medium. Immunofluorescence results showed that TGF-β1 reduced the E-cadherin expression and increased vimetin expression, whereas M2-activated microglia cell medium containing a high concentration of IL6 directly reversed this process (Figure 6A). Western blot results also showed that CC-CM could induce the MET process in 4T1-luc cells by upregulating E-cadherin and downregulating vimentin (Figure 6B). These results suggest that IL6 secreted by M2-type microglia could reprogram 4T1-luc cells through the MET process to promote early colonization in the brain. To further investigate the relationship between the JAK2–STAT3 signaling pathway and cytokine IL6, we treated 4T1-luc cells with tocilizumab, an inhibitor of IL6. As expected, the JAK2–STAT3 signaling pathway was activated by exogenous IL6, whereas tocilizumab suppressed JAK2-STAT3 activation in the presence of IL6 (Figure 6C). In summary, these results indicated that microglia pro-inflammatory M2 activation promoted the secretion of cytokine IL6, which in turn activated the JAK2–STAT3 signaling pathway in breast 4T1-luc cells.

Next, *in vitro* chemotaxis assay was used to investigate the role of chemokine CCL2 derived from CM-induced primary microglia in recruiting M-MDSCs to the brain microenvironment. The experimental procedure is shown on the left side of Figure 6D. We found that 4T1-luc CC-CM induced the migration of spleen-derived M-MDSCs across a trans-well membrane. The amount of M-MDSCs detected in the lower chamber after CM treatment was significantly increased (by 4.3-fold) (Figure 6D). To further confirm that the migration of M-MDSCs was induced by chemokine CCL2, we then neutralized CCL2 in CM using a CCL2-specific antibody (Figure 6E). As expected, a significant reduction in the migration of M-MDSCs in the lower chamber was determined after CCL2 neutralization. Altogether, our results demonstrated that M2-type microglia secreted not only CCL2 that recruited M-MDSCs but also IL6 that induced the MET process of BC cells, which could form an immunosuppressive microenvironment in the brain, and promoted the colonization of BC-BM cells.

### 3.7 β-elemene regulated the IL6/STAT3 signaling pathway and inhibited M-MDSC recruitment to inhibit brain metastasis of breast cancer

**FIGURE 7:**
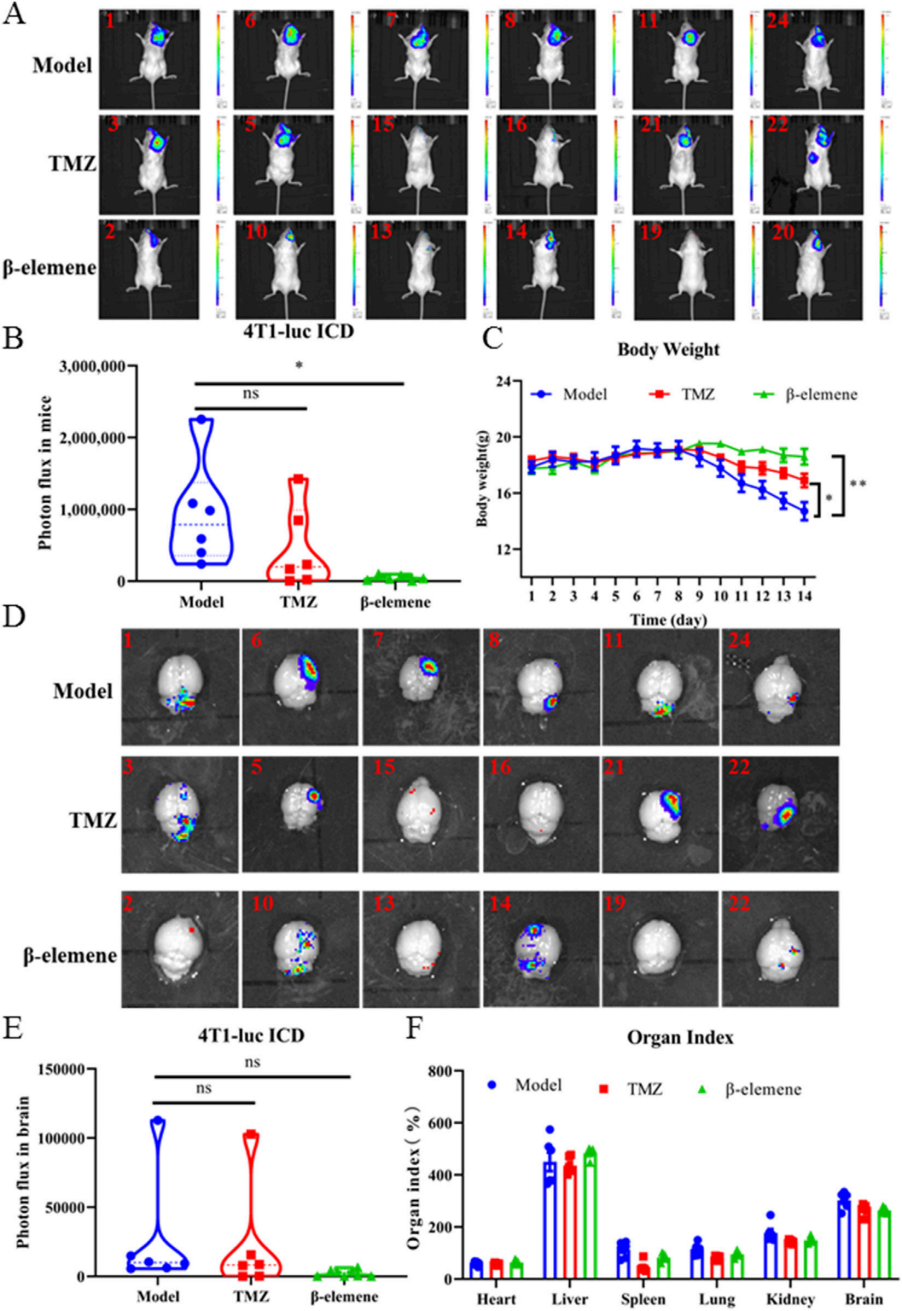
β-elemene inhibited brain metastasis of 4T1-luc breast cancer cell *in vivo*. **(A)** 4T1-luc cells brain metastases after TMZ and β-elemene treatment for 14 days determined by bioluminescence imaging. **(B)** Statistical results of *in vivo* brain tissue fluorescence intensity of each group. **(C)** Body weight of mice in each group. **(D)** Brain metastasis efficiency of 4T1-luc cells treated with TMZ and β-elemene for 14 days determined by bioluminescence imaging. **(E)** Statistical results of *in situ* brain tissue fluorescence intensity of each group. **(F)** Organ index of mice in each group. Data are mean ± SD (n = 6). Significant difference versus model group, *p < 0.05, **p < 0.01, and ***p < 0.001.

**FIGURE 8:**
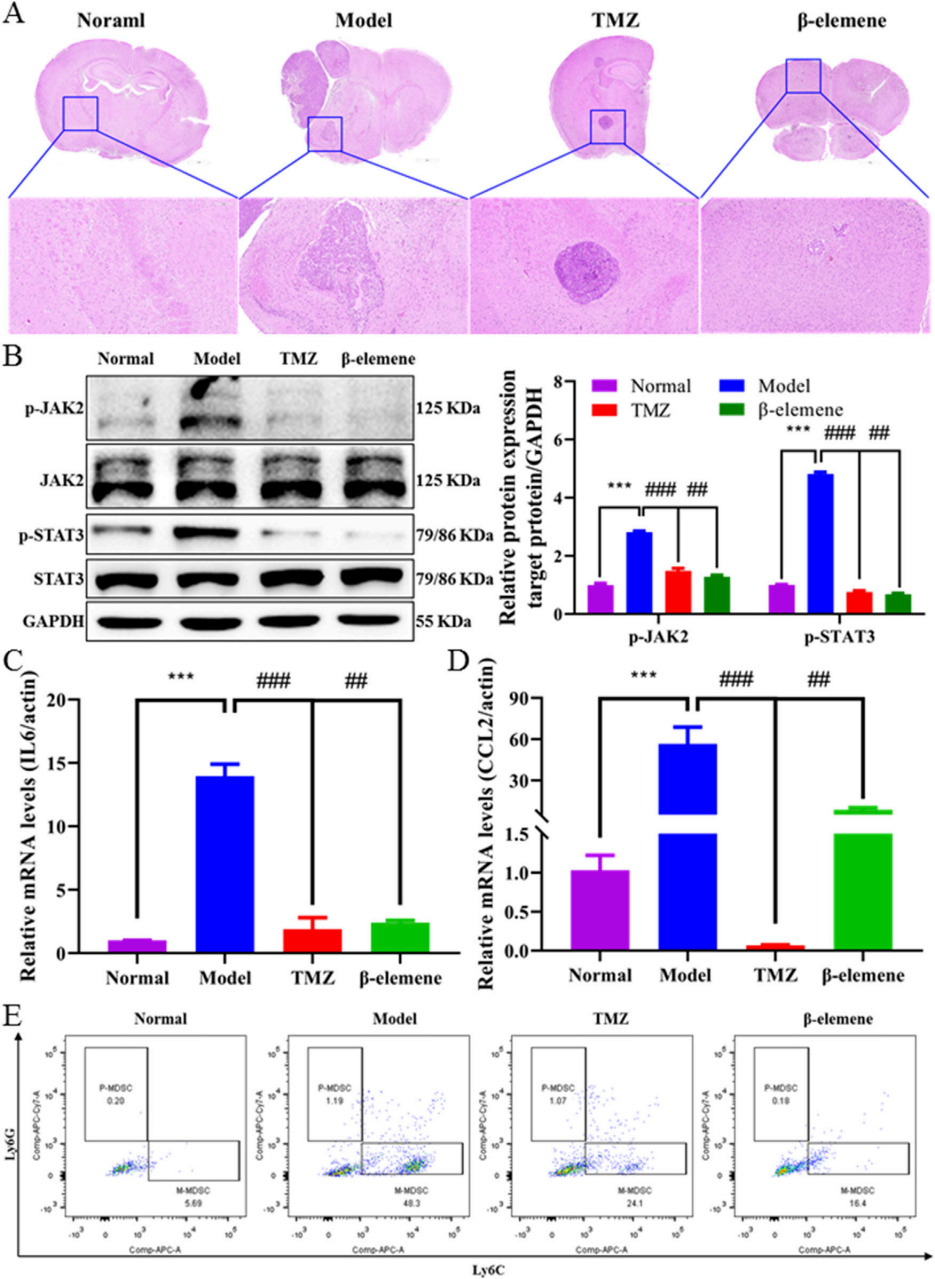
β-elemene regulated IL6/STAT3 signaling pathway and inhibited M-MDSC recruitment. **(A)** Representative images of brain HE staining treated with TMZ and β-elemene for 14 days depicted. **(B)** Western blot analysis of p-JAK2, JAK2, p-STAT3, and STAT3 in mice brain tissue treated with TMZ and β-elemene for 14 days. Relative expressions of IL6 **(C)** and CCL2 **(D)** in each group’s mice brain tissue were detected by quantitative RT-PCR. GAPDH was used to normalize gene expression. **(E)** M-MDSC and P-MDSC distributions in normal mice brain, 4T1-luc inoculation mice brain, and 4T1-luc inoculation treated with TMZ and β-elemene mice brain detected by flow cytometer. Data are mean ± SD. significant difference versus control group, ***, *P* < 0.001; significant difference versus model group, #, *p* < 0.05, ##, *p* < 0.01, and ###, *p* < 0.001.

In order to investigate whether β-elemene can prevent BC-BM, we first used the ICD *in vivo* model. As shown in Figure 7A, 4T1-luc cells were injected into the intracarotid of mice, and 3 days later, surviving mice were divided into three groups and administrated with saline, 25 mg/kg TMZ, and 100 mg/kg β-elemene. After 14 days of administration, strong fluorescence intensity was detected in the brain of mice in the control group, and body weight was significantly reduced by more than 20% (Figure 7C). The fluorescence intensity was 923,896.2667 ± 729,295.8129 in the control group, 449,049.5717 ± 568,332.177 in the TMZ group, and 41,804.48333 ± 37,218.68417 in the β-elemene group (Figure 7B). Most importantly, body weights of mice in the β-elemene group were not significantly reduced. After sacrifice, brain tissue was stripped and fluorescence intensity was measured. *In vivo* brain bioluminescence imaging results also confirmed that the mean fluorescence intensity of brain tissue in the β-elemene group was significantly reduced compared to the control group (Figures 7D,E). Organ index (Figure 7F) showed that no significant differences among three groups, indicating that β-elemene has no obvious toxic and side effects. To further explore the mechanisms of β-elemene against BC-BM, we first stained the brain tissue of each group with HE. As shown in Figure 8A, the brain tissue in the model group had been infiltrated with 4T1-luc cells, and malignant proliferating tumor cells were detected at both the edge of and inside mice brain tissue. Brain tumor volume in the TMZ group was significantly smaller than that in the model group, and the β-elemene treatment group exhibited the smallest tumor volume. The Western blot also confirmed that 4T1-luc cells could significantly activate the JAK2–STAT3 signaling pathway after metastasis to brain tissue, whereas the expression of p-JAK2 and p-STAT3 proteins in brain tissue were significantly decreased after TMZ and β-elemene treatment (Figure 8B). Meanwhile, mRNA expressions of IL6 and CCL2 in brain tissue were detected by RT-PCR. As shown in Figures 8C and D, the mRNA expressions of IL6 and CCL2 in the model group were significantly increased compared to a normal brain, whereas the mRNA levels of IL6 and CCL2 in the TMZ and β-elemene treatment groups were significantly decreased. At the same time, MDSCs in brain tissues were detected by flow cytometry. As shown in Figure 8E, M-MDSCs or P-MDSCs could barely be detected in normal brain tissue. However, after 4T1-luc cells metastasized to brain tissue, the blood–brain barrier was destroyed and there was a significant increase in MDSCs, especially M-MDSCs (43.53 ± 4.38). Brain M-MDSCs in the TMZ and ETO groups decreased to 27.77 ± 3.30 and 16.1 ± 2.67, respectively. Above all, β-elemene regulated the JAK2/STAT3 signaling pathway and inhibited M-MDSC recruitment to inhibit the BM of 4T1-luc cells.

## 4 Discussion

Improved early detection and treatment have paradoxically increased BM incidence, with 150,000–200,000 annual cases now far surpassing primary brain tumors (≈17,000) (Lamba et al., 2021). BC is one of the three most common tumors that can spread to the brain, with nearly 25% of advanced BC patients developing BMs, which can greatly reduce the quality of life and overall survival (Sun et al., 2023). Due to the unique microenvironment of the brain, it is urgent to reveal the molecular mechanisms of BC-BM and provide novel therapy strategies to improve patient prognosis. In the present study, we found that after BM of BC cells, Arg1+ microglia were significantly aggregated at the site of tumor metastasis (Figure 1F), and the levels of cytokine IL6, chemokine CCL2, were significantly increased (Figures 1D,E), suggesting an immunosuppressive microenvironment following BC-BM. *In vitro* CC-CM experiments also confirmed that BC cells (both 4T1-luc and MDA-MB-231-luc cells) could polarize microglia cells into M2 phenotype (Figure 2F) and increase the secretions of IL6 and CCL2 by microglia cells in a time-dependent manner (Figures 2D,E). Therefore, we hypothesize that once microglia adopt the M2-polarized phenotype, IL6 and CCL2 in the tumor microenvironment are mainly secreted from microglia.

In this study, we constructed two different *in vivo* mice models of BC-BM and enriched both mouse 4T1-BM2 cells and human MDA-MB-231-BM2 cells with ICT *in vivo* selection. The EMT process has been proven to play an important role in cancer metastasis; therefore, we compared the invasion and migration of BC-BMs to parent cancer cells. We found that BC-BM cells exhibited stronger migration ability (Figures 5A,B); in particular,E-cadherin (epithelial cell marker) was significantly reduced and mesenchymal cell marker vimentin was significantly increased (Figures 5E,F). TMT-based quantitative proteomics analysis revealed that there were significant differences in protein expressions between 4T1-BMs cells and parent 4T1-luc cells. In total, 503 proteins were identified as differentially expressed in 4T1-BM1 and 4T1-BM2 cells. KEGG pathway enrichment analysis revealed that the JAK–STAT signaling pathway was significantly upregulated in 4T1-BMs cells (Figure 3). Western blot results verified that the levels of both phosphorylated JAK2 and STAT3 were increased in both BC-BM1 and BC-BM2 cells, which were consistent with the proteomics results (Figures 5G,H).

However, the mesenchymal phenotype promotes the metastasis of tumor cells from the primary site while hindering the colonization of the secondary site after metastasis. Therefore, the process of MET in metastatic tumor cells is a necessary process affecting early colonization (Dongre and Weinberg, 2019; Pei et al., 2019). We then explored whether the immunosuppressive microenvironment created by M2 microglia promotes the MET process in BM tumor cells. We first triggered the EMT process in 4T1-luc cells *via* TGF-β1 and then treated with CC-CM to observe the MET process of 4T1-luc cells. We found that CC-CM could reverse the decreased expression of E-cadherin and the increased expression of vimentin induced by TGF-β1 (Figures 6A,B). We also found that MDSCs, especially M-MDSCs, were significantly increased in BC-BM brain tissue (Figure 4F). An *in vitro* chemotaxis assay also confirmed that CCL2-specific antibody significantly inhibited the efficacy of M-MDSC recruitment (Figures 6D,E).

TCM has been used to treat human disease for thousand years in China, and it plays an important role in the prevention and treatment of numerous diseases (Zhou et al., 2022). EZ exhibits many pharmacological activities, including anti-inflammatory and analgesic, which has been widely used in clinics. Modern pharmacological studies have confirmed that EZO has a broad spectrum of antitumor activities and could significantly inhibit the growth of a variety of malignant tumors. β-elemene is the main pharmacodynamic component of EZO; in order to study the effect of β-elemene on BC-BM, the ICD model was used for *in vivo* validation . As expected, after 14 days of administration, both *in situ* and *in vivo* bioluminescence imaging results confirmed that the mean fluorescence intensity of brain tissue in the β-elemene group was significantly decreased compared with the control group (Figures 7A,D). Most importantly, the body weights of mice in the β-elemene group did not show significant reduction, implying that β-elemene did not exert significant adverse effects (Figure 7C). HE staining results showed that tumor cell infiltration in the β-elemene group was significantly less compared to that in the model group (Figure 8A). Further mechanism studies confirmed that β-elemene regulated the JAK2/STAT3 signaling pathway (Figure 7B) and inhibited M-MDSC recruitment (Figure 7E) to inhibit BC-BM.

In summary, our results indicate that the process of BC cell colonization and growth in brain tissue is as follows. First, BC cells undergo an EMT process, which promotes BC cells to enter the blood circulation and reach the brain tissue, where BC cells could promote the M2-type polarization of microglia and secrete IL6 and CCL2. Cytokine IL6 promotes the MET process of BC cells to accelerate colonization in the brain tissue. Chemokine CCL2 recruits M-MDSCs to form an immunosuppressive brain microenvironment and further promotes the growth of BC cells in brain tissue. Our results also showed that BC-BMs cells activated the M2 polarization of microglia and secreted IL6 and CCL2. Subsequently, microglia-derived IL6 could promote BC-BM early colonization by inducing the MET process. Meanwhile, microglia-derived CCL2 could promote the growth of BC-BMs in the brain by recruiting M-MDSCs to form an immunosuppressive microenvironment. β-elemene, a pharmacodynamic component of EZO, could significantly inhibit BC-BM in mice established by the ICD model by regulating the IL6/STAT3 signaling pathway and suppressing M-MDSC recruitment. Our study relies on mice xenograft models, which fail to fully recapitulate the complex microenvironmental interactions of human brain metastasis. In the future, we will use syngeneic and humanized mouse models, integrate multi-omics technologies to dissect brain metastasis heterogeneity, and develop precise therapeutic strategies targeting tumor–microenvironment crosstalk. Overall, our results further improve the theoretical concepts of tumor cell colonization and growth in the brain microenvironment.

## Data Availability

The original contributions presented in the study are included in the article/Supplementary Material; further inquiries can be directed to the corresponding authors.

## Abbreviations

BC, breast cancer; BM, brain metastasis; BC-BM, breast cancer brain metastasis; MSCS, mesenchymal stem cells; EMT, epithelial-to-mesenchymal transition; MET, mesenchymal-to-epithelial transition; TCM, traditional Chinese medicine; EZO, Curcuma Rhizome oil; TMZ, temozolomide; ICT, intracardiac; ICD, intracarotid; CM, conditioned media; MG, primary microglia cells; PCA, principal component analysis; KEGG, Kyoto Encyclopedia of Genes and Genomes; BP, biological processes; CC, cell components; MF, molecular functions; CC-CM, co-culture conditioned medium; MDSCs, myeloid-derived suppressor cells; M-MDSCs, monocytic myeloid-derived suppressor cells; P-MDSCs, polymorphonuclear myeloid-derived suppressor cells; SD, standard deviation.

## References

[B1] Akhtar M. Haider A. Rashid S. Al-Nabet A. D. M. H. (2019). Paget's “seed and soil” theory of cancer metastasis: an idea whose time has come. Adv. Anat. Pathol. 26, 69–74. 10.1097/Pap.0000000000000219 30339548

[B2] Bailleux C. Eberst L. Bachelot T. (2021). Treatment strategies for breast cancer brain metastases. Brit J. Cancer 124, 142–155. 10.1038/s41416-020-01175-y 33250512 PMC7782834

[B3] Barnholtz-Sloan J. S. Sloan A. E. Davis F. G. Vigneau F. D. Lai P. Sawaya R. E. (2004). Incidence proportions of brain metastases in patients diagnosed (1973 to 2001) in the metropolitan Detroit cancer surveillance system. J. Clin. Oncol. 22, 2865–2872. 10.1200/Jco.2004.12.149 15254054

[B4] Bray F. Laversanne M. Sung H. Y. A. Ferlay J. Siegel R. L. Soerjomataram I. (2024). Global cancer statistics 2022: GLOBOCAN estimates of incidence and mortality worldwide for 36 cancers in 185 countries. CA Cancer J. Clin. 74, 229–263. 10.3322/caac.21834 38572751

[B5] Brosnan E. M. Anders C. K. (2018). Understanding patterns of brain metastasis in breast cancer and designing rational therapeutic strategies. Ann. Transl. Med. 6, 163. 10.21037/Atm.2018.04.35 29911111 PMC5985267

[B6] Caffarel M. M. Braza M. S. (2022). Microglia and metastases to the central nervous system: victim, ravager, or something else? J. Exp. Clin. Canc Res. 41, 327. 10.1186/s13046-022-02535-7 PMC9677912 36411434

[B7] Cagney D. N. Martin A. M. Catalano P. J. Redig A. J. Lin N. U. Lee E. Q. (2017). Incidence and prognosis of patients with brain metastases at diagnosis of systemic malignancy: a population-based study. Neuro-Oncology 19, 1511–1521. 10.1093/neuonc/nox077 28444227 PMC5737512

[B8] Chen X. Huang C. Li K. Liu J. Zheng Y. Feng Y. (2023). Recent advances in biosynthesis and pharmacology of β-elemene. Phytochem. Rev. 22, 169–186. 10.1007/s11101-022-09833-0

[B9] Chen Y. N. Hu M. R. Wang L. Chen W. D. (2020). Macrophage M1/M2 polarization. Eur. J. Pharmacol. 877, 173090. 10.1016/J.Ejphar.2020.173090 32234529

[B10] Dongre A. Weinberg R. A. (2019). New insights into the mechanisms of epithelial-mesenchymal transition and implications for cancer. Nat. Rev. Mol. Cell Bio 20, 69–84. 10.1038/s41580-018-0080-4 30459476

[B11] Feng Y. Hu X. Q. Zhang Y. R. Wang Y. (2024a). The role of microglia in brain metastases: mechanisms and strategies. Aging Dis. 15, 169–185. 10.14336/Ad.2023.0514 37307835 PMC10796095

[B12] Feng Y. W. An Q. W. Zhao Z. Q. Wu M. T. Yang C. Q. Liang W. Y. (2024b). Beta-elemene: a phytochemical with promise as a drug candidate for tumor therapy and adjuvant tumor therapy. Biomed. Pharmacother. 172, 116266. 10.1016/J.Biopha.2024.116266 38350368

[B13] Fidler I. J. (2003). The pathogenesis of cancer metastasis: the 'seed and soil' hypothesis revisited. Nat. Rev. Cancer 3, 453–458. 10.1038/nrc1098 12778135

[B14] Gao T. H. Liao W. Lin L. T. Zhu Z. P. Lu M. G. Fu C. M. (2022). Curcumae rhizoma and its major constituents against hepatobiliary disease: pharmacotherapeutic properties and potential clinical applications. Phytomedicine 102, 154090. 10.1016/j.phymed.2022.154090 35580439

[B15] Giaquinto A. N. Sung H. Newman L. A. Freedman R. A. Smith R. A. Star J. (2024). Breast cancer statistics 2024. Ca-Cancer J. Clin. 74, 477–495. 10.3322/caac.21863 39352042

[B16] Hosonaga M. Saya H. Arima Y. (2020). Molecular and cellular mechanisms underlying brain metastasis of breast cancer. Cancer Metast Rev. 39, 711–720. 10.1007/s10555-020-09881-y PMC7497304 32399646

[B17] Huang Y. H. Hong W. Q. Wei X. W. (2022). The molecular mechanisms and therapeutic strategies of EMT in tumor progression and metastasis. J. Hematol. Oncol. 15, 129. 10.1186/s13045-022-01347-8 36076302 PMC9461252

[B18] Karimpour M. Ravanbakhsh R. Maydanchi M. Rajabi A. Azizi F. Saber A. (2021). Cancer driver gene and non-coding RNA alterations as biomarkers of brain metastasis in lung cancer: a review of the literature. Biomed. Pharmacother. 143, 112190. 10.1016/J.Biopha.2021.112190 34560543

[B19] Kuzminska J. Szyk P. Mlynarczyk D. T. Bakun P. Muszalska-Kolos I. Dettlaff K. (2024). Curcumin derivatives in medicinal chemistry: potential applications in cancer treatment. Mol. (Basel, Switzerland) 29, 5321. 10.3390/molecules29225321 PMC11596437 39598712

[B20] Lamba N. Wen P. Y. Aizer A. A. (2021). Epidemiology of brain metastases and leptomeningeal disease. Neuro-Oncology 23, 1447–1456. 10.1093/neuonc/noab101 33908612 PMC8408881

[B21] Lim M. Fletcher N. McCart Reed A. Flint M. Thurecht K. Saunus J. (2022). Modeling brain metastasis by internal carotid artery injection of cancer cells. J. Vis. Exp. 10.3791/64216 35993751

[B22] Lin L. T. Zhou X. M. Gao T. H. Zhu Z. P. Qing Y. Liao W. (2024). Herb pairs containing Curcumae Rhizoma (Ezhu): a review of bio-active constituents, compatibility effects and t-copula function analysis. J. Ethnopharmacol. 319, 117199. 10.1016/J.Jep.2023.117199 37844744

[B23] Liu J. Y. Geng X. F. Hou J. X. Wu G. S. (2021). New insights into M1/M2 macrophages: key modulators in cancer progression. Cancer Cell Int. 21, 389. 10.1186/s12935-021-02089-2 34289846 PMC8296555

[B24] Mizutani R. Hirata E. (2024). Intracardiac injection mouse model to study cancer cell dormancy in brain metastasis. Methods Mol. Biol. Clift. N.J. 2811, 113–122. 10.1007/978-1-0716-3882-8_8 39037653

[B25] Nayak D. Roth T. L. McGavern D. B. (2014). Microglia development and function. Annu. Rev. Immunol. 32, 367–402. 10.1146/annurev-immunol-032713-120240 24471431 PMC5001846

[B26] Orecchioni M. Ghosheh Y. Pramod A. B. Ley K. (2019). Macrophage polarization: different gene signatures in M1(LPS+) vs. Classically and M2(LPS-) vs. Alternatively activated macrophages. Front. Immunol. 10, 1084. 10.3389/Fimmu.2019.01084 31178859 PMC6543837

[B27] Pei D. Q. Shu X. D. Gassama-Diagne A. Thiery J. P. (2019). Mesenchymal-epithelial transition in development and reprogramming. Nat. Cell Biol. 21, 44–53. 10.1038/s41556-018-0195-z 30602762

[B28] Rathi J. Kumar S. Sindhu R. K. Dhiman A. Faujdar S. (2024). Pharmacognostical characterization, GC-MS profiling, and elemental analysis of Curcuma caesia Roxb. rhizomes for public health. J. complementary Integr. Med. 21, 360–369. 10.1515/jcim-2024-0151 38940214

[B29] Sun H. N. Xu J. N. Dai S. Ma Y. W. Sun T. (2023). Breast cancer brain metastasis: current evidence and future directions. Cancer Med-Us 12, 1007–1024. 10.1002/cam4.5021 PMC9883555 35822637

[B30] Tan T. T. Li J. Luo R. H. Wang R. R. Yin L. Y. Liu M. M. (2021). Recent advances in understanding the mechanisms of elemene in reversing drug resistance in tumor cells: a review. Mol. 26, 5792. 10.3390/Molecules26195792 PMC8510449 34641334

[B31] Wu Y. Q. Tong T. (2022). Curcumae Rhizoma: a botanical drug against infectious diseases. Front. Pharmacol. 13, 1015098. 10.3389/fphar.2022.1015098 36703758 PMC9871392

[B32] Xie Q. Li F. Fang L. Liu W. Gu C. (2020). The antitumor efficacy of β-elemene by changing tumor inflammatory environment and tumor microenvironment. BioMed Res. Int. 2020, 6892961. 10.1155/2020/6892961 32149121 PMC7054771

[B33] Xu W. W. Patel N. Deng Y. X. Ding S. Wang T. Y. Zhang H. J. (2023). Extracellular vesicle-derived LINC00482 induces microglial M2 polarization to facilitate brain metastasis of NSCLC. Cancer Lett. 561, 216146. 10.1016/J.Canlet.2023.216146 36963460

[B34] Yang J. Antin P. Berx G. Blanpain C. Brabletz T. Bronner M. (2020). Guidelines and definitions for research on epithelial-mesenchymal transition. Nat. Rev. Mol. Cell Bio 21, 341–352. 10.1038/s41580-020-0237-9 32300252 PMC7250738

[B35] Zhang C. Y. Lowery F. J. Yu D. H. (2017). Intracarotid cancer cell injection to produce mouse models of brain metastasis. J Vis. Exp., 55085. 10.3791/55085 28287553 PMC5409267

[B36] Zhou J. B. Wang L. Peng C. Peng F. (2022). Co-targeting tumor angiogenesis and immunosuppressive tumor microenvironment: a perspective in ethnopharmacology. Front. Pharmacol. 13, 886198. 10.3389/Fphar.2022.886198 35784750 PMC9242535

[B37] Zhou Y. Xie M. Song Y. Wang W. Zhao H. Tian Y. (2016). Two traditional Chinese medicines Curcumae radix and Curcumae rhizoma: an ethnopharmacology, phytochemistry, and pharmacology review. Evidence-based complementary Altern. Med. 2016, 4973128. 10.1155/2016/4973128 PMC4775794 27057197

[B38] Zhu X. Quan Y. Y. Yin Z. J. Li M. Wang T. Zheng L. Y. (2023). Sources, morphology, phytochemistry, pharmacology of Curcumae longae rhizoma, Curcumae radix, and Curcumae rhizoma: a review of the literature. Front. Pharmacol. 14, 1229963. 10.3389/fphar.2023.1229963 37719857 PMC10500466

